# A Bibliometric Analysis on Global Psychological and Behavioral Research Landscape on COVID-19 Pandemic

**DOI:** 10.3390/ijerph19020879

**Published:** 2022-01-13

**Authors:** Xilu Dong, Xuqiu Wei, Fei Shu, Qiang Su, Juntao Wang, Ning Liu, Junping Qiu

**Affiliations:** 1 Chinese Academy of Sciences and Education Evaluation, Hangzhou Dianzi University, #1158, Block 2, Baiyang Street, Qiantang New District, Hangzhou 310018, China; fei.shu@mail.mcgill.ca (F.S.); liuning@hdu.edu.cn (N.L.); jpqiu@hdu.edu.cn (J.Q.); 2 Institute of Information Management, Shandong University of Technology, 266 Xincun West Road, Zhangdian, Zibo 255000, China; 3 Zhejiang Academy of Higher Education, Hangzhou Dianzi University, #1158, Block 2, Baiyang Street, Qiantang New District, Hangzhou 310018, China; sq@hdu.edu.cn; 4 Faculty of Engineering, Architecture & Information Technology, The University of Queensland, St Lucia, QLD 4072, Australia; juntao.wang@uqconnect.edu.au

**Keywords:** COVID-19, psychological, behavioral, bibliometric, VOSviewer

## Abstract

The COVID-19 pandemic outbreak in December 2019 has spread globally. The ongoing psychological and behavioral effects of the COVID-19 pandemic, which poses a major challenge to humanity, are of concern to researchers. To understand the academic community’s attention, focus and research collaboration on psychological and behavioral research during the COVID-19 pandemic, we conducted a macro analysis using a bibliometric approach. Using the topic selection strategy of TS = (“COVID-19” OR “coronavirus disease 2019” OR “SARS-CoV-2” OR “2019-nCoV”) AND TS = (“behavio*”) AND TS = (“psycholog*”), 2096 high-quality research articles and reviews were downloaded as data from the Web of Science core collection on 16 November 2021. Through analysis and visualization, the following conclusions are drawn in this study: (1) The popularity and importance of psychological and behavioral research under COVID-19 has increased significantly and needs further attention; (2). Related research focuses on eight hotspots, with quarantine, health care workers, the elderly, students, pregnant women, family, consumers, social media and emergency preparedness knowledge as the focus of the research object; and (3) Research collaboration is relatively high at the author, organizational and national levels. However, low-income countries need to get more attention. Furthermore, this article would help researchers make decisions for the research of psychological and behavioral issues under COVID-19 and planning for future prospects to contribute to academic development and applied methodology.

## 1. Introduction

The COVID-19 pandemic that broke out at the end of 2019 has developed into a global pandemic and become the world’s largest pandemic since the Spanish flu in 1918 [1]. The pandemic affects people’s daily lives in many areas [2,3,4]. However, these impacts are not only caused by COVID-19 itself, but also due to measures such as the lockdown and quarantines, the expansion of social distance, and classification and management of health QR codes in response to the pandemic [5,6,7], which paused many activities. Some literature has demonstrated that these measures have an obvious effect on COVID-19 pandemic prevention and control [8,9,10]. However, together with the pandemic itself, they have caused people’s psychological and behavioral problems, and people’s psychological recovery from the pandemic is far more difficult than disease treatment [11]. Moreover, psychological health issues may hinder the advancement of COVID-19 pandemic prevention and control to a certain extent, especially when fake news and false information are disseminated during the pandemic [12]. Whether it is now or in the future, people’s psychological and behavioral health under the COVID-19 pandemic is an area that deserves further attention. So, what are the focus of the psychological and behavioral research on the COVID-19 pandemic in academia? And what is the status of research collaboration in this field of research? Given that the COVID-19 pandemic is a typical sudden large-scale public health event, answering these questions will help reveal the characteristics of psychological and behavioral research during the pandemic and provide some references for further research and collaboration in the academic community.

## 2. Literature Review

The Severe Acute Respiratory Syndrome (SARS) incident has made us aware that acute infectious diseases can cause psychological problems such as inattention, nervousness, irritability, anxiety, depression and post-traumatic stress [13,14]. Since COVID-19 is more persistent and widespread than SARS, its impact on people’s daily lives is also more extensive, and people’s psychological and behavioral problems occur more frequently than before [15,16]. A lot of evidence can be obtained through empirical research [17,18,19,20]. At the earliest, the psychological health problems of medical staff on the front line of this crisis attracted the attention of researchers. A survey found that a considerable proportion of health care workers experienced emotional and sleep disorders during the pandemic [21]. Based on research on economic recession, social distancing, and school closures, Liu [22] found that the COVID-19 pandemic has weakened or lacked the social support system of teenagers, affecting their normal psychological development. In addition to young people and medical staff, the psychological health of the elderly is also one of the areas of research focus during the pandemic. Such groups need to get more attention, and they may have more serious panic in the period of the pandemic. Jiang [23] believes that the panic was caused by the social and psychological consequences of people’s information shortage about the virus and the pandemic. From the perspective of information asymmetry, the elders are more likely to become vulnerable groups. Since this crisis involves large-scale behavior changes and imposes a huge psychological burden on individuals, Van Bavel et al. [24] believe that insights from social and behavioral science can be used to make human behavior conform to the recommendations of epidemiologists and public health experts.

Since the outbreak of the pandemic, scientific research on COVID-19 has been growing exponentially. This surge in numbers has spawned literature on the use of methods in bibliometrics by researchers in different fields to study COVID-19 [25,26,27,28,29,30,31,32], providing some references and directional guidance for further research regarding the psychological and behavioral research under the COVID-19 pandemic [33,34]. Al-Jabi [35] studied hotspots of depression research of COVID-19 from a global perspective. Although this was the first study to describe and visualize scientific research on COVID-19 and its effect on depression, psychological problems other than depression were excluded from the study. Soytas [36] pointed out that the elders need more social and psychological support due to the fatality rate, social isolation, dementia, loneliness and other reasons. A recent study published by Wang [37] and his co-authors used high-frequency keywords analysis in the research field of COVID-19 and psychological research. The research was limited to Chinese literature, which provides very limited references for international researchers. From the publication of the above-mentioned related research to the launch of the current study, there were a large number of publications in between. We tried to conduct a quantitative analysis of the psychology- and behavior-related research in the past two years by using the latest data of Web of Science (WoS) and more extensive psychological and behavioral research and exploration, calling on experts, relevant staff and authoritative departments to devote more attention and practical actions to this field in the post-pandemic era. In addition, this article will also help the young researcher to find potential countries, institutes and researchers for further studies or collaboration.

## 3. Methodology

### 3.1. Data Source and Search Strategies

WoS is known as one of the most influential online databases [38,39] largely due to its bibliometric and visual research. To have relevant research articles in the WoS database, the selection of publications was done prudently, and only research articles and reviews were retrieved from the core collection of WoS, which includes the Science Citation Index Expanded (SCI-E), Social Sciences Citation Index (SSCI), Arts & Humanities Citation Index (AHCI), Conference Proceedings Citation Index-Science (CPCI-S), Conference Proceedings Citation Index-Social Science & Humanities (CPCI-SSH), Emerging Sources Citation Index (ESCI), Current Chemical Reactions (CCRE), and Index Chemicus (IC). We used an advanced search strategy of topic search, and the query was TS = (“COVID-19” OR “coronavirus disease 2019” OR “SARS-CoV-2” OR “2019-nCoV”) AND TS = (“behavio*”) AND TS = (“psycholog*”). To avoid possible bias produced by continuous database updating, the retrieval and export of documents were created within one day (16 November 2021). After further examination of the retrieved data, we omitted documents that had been indexed as an erratum. For instance, we reviewed three articles published in 2019 and found that they were not related to this current pandemic and hence were removed from the analysis. The first published study in the psychological and behavioral literature on COVID-19 was published in 2020 [40]. A total of 2096 articles directly and indirectly linked with the psychological and behavioral aspects of COVID-19 were exported in the plain text file after using a topic search strategy, and the record content was a full record. The following bibliometric parameters were included: publication type, title, authorship, abstract, keywords, institution, country/region, journal, and citation frequency. It is worth mentioning that when we retrieved COVID-19 related articles on the same day, the results showed that there were more than 30 related documents that had been published in 2019, which indicates that research in the field of psychology and behavior is lagging as a subtopic of COVID-19. The details of the data collection are summarized in Table 1.

### 3.2. Bibliometric Tool and Functions

VOSviewer software was used to construct network visualization maps [41]. The tool was used to analyze the network map of co-occurrence of high-frequency terms (based on keywords) with scaling algorithms, and identify them into different clusters and color them simultaneously. By using the function of co-occurrence analysis of terms, we can quickly capture the hotspots related to COVID-19 and psychological and behavioral research. By counting the frequency of a term appearing in keywords of the selected documents, we could determine the value of terms. The minimum number of co-occurrence terms is 20 in the presenting research. In addition, VOSviewer will evaluate the collaborative relationships between countries/regions, authors, and institutions. For more information about other functions of VOSviewer, please refer to the article by van Eck and Waltman [42].

## 4. Results

Among 2096 original research articles and reviews included in this study, 578 were published in 2020, 1500 were published in 2021, and 18 early access articles are marked as published in 2022. 1262 out of the 2096 papers were cited, with a total of 17,832 citations and an average of 8.5 citations. Citation counts are generally considered a measure of utilization and contribution to published articles [43] so that they reflect the importance and influence of articles in the academic community [44]. WoS describes a highly cited paper in COVID-19 and psychological and behavioral research as follows: as of May/June 2021, the highly cited paper has received enough citations to place it in the top 1% of its academic field, based on the high citation threshold for the field and publication year. 132 were labeled as highly cited papers, accounting for 6.3% of all articles. In the current study we only consider the count obtained on the day of download, although the citation frequency of these articles may increase as more articles are published. The average citation rate of all highly cited papers was 73.6, among which 20 articles were cited 100 times or more. In general, this field has received enough attention from the academic community, and these numbers made it a reality for us to analyze the research focus and collaboration status using bibliometric methods.

### 4.1. Research Focus

Combining the frequency of keywords and the clusters formed between them, we can form an understanding of psychological and behavioral research topics in the context of the COVID-19 pandemic. Considering that some keywords express the same meaning, it is necessary to preprocess the data, that is, to merge synonyms. First, we imported the downloaded data into the BibExcel software and extracted the keywords provided by the author. A total of 4714 keywords were detected, with a total frequency of 11,473 occurrences. Next, synonyms were replaced by the same keyword, for example, “COVID-19”, “coronavirus disease 2019”, “SARS-CoV-2”, “2019-nCoV” and other synonyms were unified as “COVID-19”. In another example, “fear of COVID-19”, “fear for COVID-19”, “the fear of COVID-19” and “fears of COVID-19” were unified as “fear of COVID-19”. The purpose of this processing is to make the frequency of each keyword accurate. In the end, we got 4130 different keywords. The data was imported into VOSviewer, and all keywords were analyzed for co-occurrence. The minimum total link strength of a keyword was set to 20, and 256 keywords meet the threshold. Undoubtedly, “COVID-19” is the most frequent occurrence of a keyword. Considering “COVID-19” appears in the greatest number of documents, it is not necessary to analyze their co-occurrence. In the end, 255 high-frequency co-occurrence keywords were obtained. The keyword with the strongest total link strength was “mental health”, which reached 931. “Mental health” and the other nine keywords rank the top 10 in total link strength (see Table 2).

**Figure 1:**
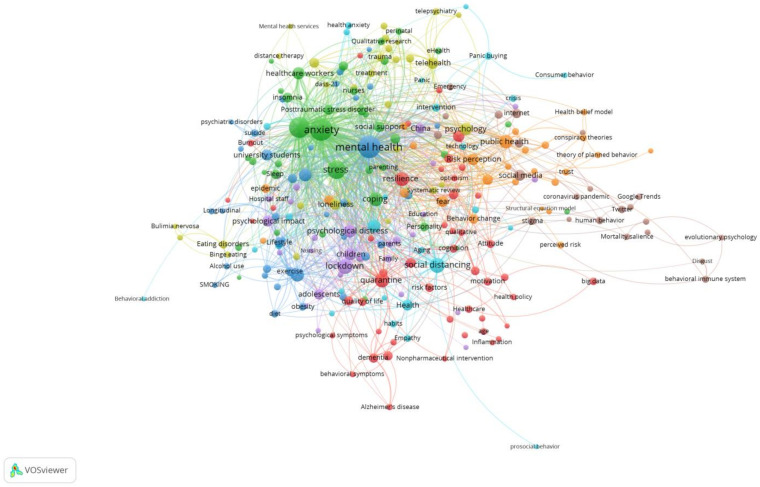
Network map of keywords visualization for publishing articles.

For each of the 255 keywords, the total strength of the co-occurrence links with other keywords was calculated, and the keywords with the greatest total link strength were selected. Figure 1 is a co-occurrence map of keywords in the articles related to psychological and behavioral research in the context of the COVID-19 pandemic. Among them, the normaliztion presentation method is “*LinLog/modularity*”, and the minimum cluster size is 20. The eight colors represent different thematic clusters, which are the focus of this research field. The size of each circle reflects the co-occurrence frequency of the marked keywords, that is, the larger the circle, the higher the frequency. The largest cluster is the red one, with a total of 48 high-frequency co-occurrence keywords. The green cluster and the dark blue cluster have 37 and 36 co-occurrence keywords, respectively. The yellow cluster and the purple cluster have 30 and 29 co-occurrence keywords, respectively. The light blue cluster and the orange cluster have 27 and 26 co-occurrence keywords, respectively. The smallest brown cluster on the right has 22 co-occurrence keywords. The high-frequency keywords that form each cluster, such as “anxiety”, “stress” and “coping” in the green cluster, can be found quickly.

### 4.2. Research Collaboration

**Figure 2:**
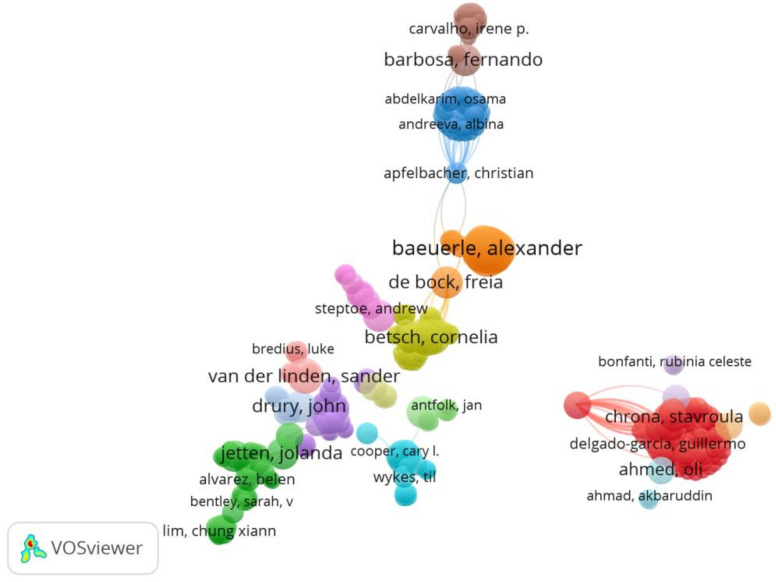
Network map of author collaboration visualization.

Co-authorship is one of the types of analysis in VOSviewer, and this kind of function includes three units of analysis, these being “authors”, “organizations” and “countries”. VOSviewer was used to extract all the authors, and a total of 10,762 were obtained. On average, each article has at least five authors. When we set the minimum number of documents of an author to two, only 891 meet the threshold, and when we set the minimum number of documents of an author to five, only 44 met the threshold (see Table 3). For each of the 10,762 authors, the total strength of the co-authorship links with other authors was calculated. The authors with the greatest total link strength were selected. Authors among the 2096 papers were assessed in terms of research collaboration with a minimum contribution of one article. However, some of the 10,762 authors are not connected. The largest set of connected authors consists of 588 authors. The clusters are shown in Figure 2.

**Figure 3:**
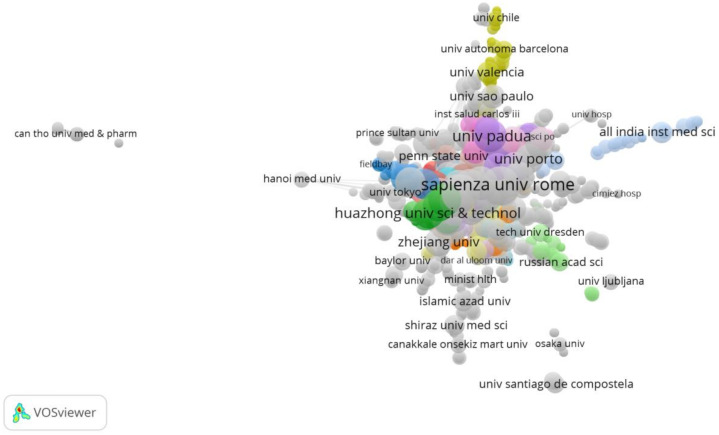
Network map of institution collaboration visualization.

Setting “organizations” as the analysis unit, a total of 3481 were extracted, and they contributed to at least one paper. When we set the minimum number of documents of an organization to two, five, and 10 in turn, there are 996, 259, and 67 papers that meet the thresholds, respectively. When the minimum number of documents of an organization is set to 15, only 27 organizations meet the thresholds (see Table 4). Organizations were assessed in research collaboration with a minimum contribution of one article. However, some of the organizations are not connected. The largest set of connected organizations consists of 2775 organizations. The clusters are shown in Figure 3.

**Figure 4:**
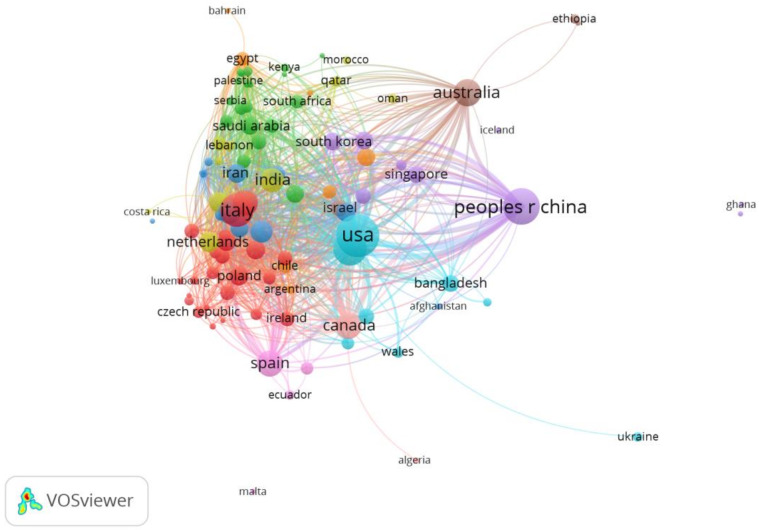
Network map of country collaboration visualization.

Finally, we set “countries” as the analysis unit, and a total of 109 countries and regions are counted. When we set the minimum number of documents of a country to five, 10, and 20, respectively, and 70, 55, and 36 met the thresholds, respectively (see Table 5). Countries and regions were assessed in research collaboration with a minimum contribution of one article. However, a few numbers of the countries and regions are not connected. The largest set of connected countries and regions consists of 100 countries. The clusters are shown in Figure 4.

## 5. Discussion and Limitation

Although articles on psychological and behavioral research under COVID-19 have grown rapidly in the past two years, when we searched COVID-19-related publications using the same database, we obtained as many as 148,201 related articles and reviews. This shows that articles on psychological and behavioral research only account for 1.4% of COVID-19 research, which is a very small portion. Moreover, the most cited COVID-19-related literature was published by Huang Chaolin and his co-authors [45]. We retrieved 19,804 citations to this article on 16 November 2021. Among highly cited papers on psychological and behavioral research under COVID-19, the most cited paper was published by Van Bavel and his co-authors [24]; it has been cited 1219 times. However, we still get some research hotspots through the co-occurrence clustering of keywords and obtain some knowledge of research collaboration through the visualization maps.

The eight-color clusters in Figure 1 focus on the following. The first is the impact of quarantine policies and measures on people, which have significantly affected people’s quality of life and subjective well-being. For example, the habit of wearing a mask can indeed prevent the spread of the virus to a large extent, but it tests people’s compliance with this behavior. In addition, the challenge of quarantine to the elderly is particularly prominent. Because of the elderly’s advanced age, and since many of them suffer from Alzheimer’s disease or behavioral and psychological symptoms of dementia, once they are quarantined, the feeling of despair is very likely to be amplified. In this way, the mental health and behavior of caregivers may deserve more attention because they have also endured greater challenges and responsibilities than ever before. The second is the manifestation of psychological and behavioral problems caused by the pandemic. Judging from the frequency of keywords, anxiety, depression, and stress are high-incidence psychological problems. In addition, the pandemic has also produced symptoms such as worry, distress, insomnia, alcoholism, self-isolation, and post-traumatic stress disorder. Due to the differences in people’s psychological resilience, different people need different levels of social support when coping with the pandemic. Among them, medical staff, students, and pregnant women have become the key research objects of this topic. The third is the research on the mental health of young people, especially university students. The knowledge map shows that such groups have suicidal tendencies and behaviors. Their psychological and behavioral problems arise from three main dimensions: their lifestyle, the change in learning methods, and the reduction in physical activity. In addition to changes in diet and work and rest, their interaction with teachers, classmates and friends has also been greatly reduced. Some international students also face long-term separation from their families, resulting in great constraints on emotional sustenance. In addition, online teaching poses a lot of challenges to students’ autonomous learning abilities. Finally, lack of physical activity may lead to some unhealthy habits, such as overeating, sitting for a long time, smoking, and using electronic devices for extended periods of time.

The fourth is to study how to break through the limitations of the blockade to try to solve the public’s mental health problems. During the pandemic, people’s ability to obtain professional treatment has been limited, but new attempts are constantly being explored. On the one hand, big data and the Internet provide convenience for remote treatment. On the other hand, people need to manage themselves. For example, one can systematically evaluate his or her mental health through the results of the DASS-21, and feed it back to experts or institutions that can provide mental health services through the Internet to seek appropriate help. The fifth is the psychological and behavioral research on the family. The lockdown policy puts parents under more pressure in taking care of their children. Especially for families with children with autism or attention deficit hyperactivity disorder, when facing public health emergencies like COVID-19, it seems that it takes more experience to manage psychological distress and negative emotions. The sixth is to explore the psychology and behavior of consumers in the context of social isolation. The mutation of viruses and the limited effects of vaccines have brought uncertainty and insecurity to the public. The willingness to ensure well-being in the age of risk has given birth to people’s tendency towards the consumer behavior of panic buying. People do not want the empty scenes that they see when they enter the supermarket to happen to them. Even if travel is restricted for a long time, at least material needs must be guaranteed. Hoarding some materials can resist unknown risks to a certain extent; however, it is not known whether this kind of panic buying behavior will damage the rationality of consumption. The seventh is the development of emergency preparedness knowledge in this field. The pandemic has adjusted people’s attitudes, emotions, cognitions, and trust mechanisms between people by affecting all aspects of people’s lives. Changes and accidents seem to have become the norm. Keywords such as “preventive behavior”, “theory of planned behavior”, “health belief model” and “health communication” indicate that the pandemic may have helped people increase their knowledge of emergency preparedness to varying degrees. Pre-education can help shape behavior, and it can also enhance the public’s understanding of protective behavior in disaster events [46,47]. The last cluster indicates the need to pay attention to the psychological and behavioral effects of social media. We believe that in the face of new mutant viruses that may emerge in the future it is necessary to disseminate enough knowledge about how to face the pandemic to the public to increase the public’s hope and confidence in life. In the case of false information spreading throughout the world, social media tends to spread terrorist information and cause group panic. In addition, information monitoring is very common and intense, and it may be necessary to worry that some people will feel shame when information literacy is not balanced.

Based on the above visualization maps, we may be able to quickly find important authors, organizations and countries or regions in this research field. Most of the authors only appeared in one article, indicating that the authors cooperate extensively. Due to the differences in the pandemic situation, national policies and measures in different countries, if a global force against the pandemic is to be formed, then the joint completion of a specific study by multiple researchers will help in consensus formation. This high degree of collaboration can be found in highly cited papers. In Van Bavel and his co-authors’ article [24], forty-three experts highlighted the need and urgency of social and behavioral science for an effective response to the COVID-19 pandemic. Moreover, the earliest highly cited paper, which was cited 69 times, was published by Yuan and six co-authors [48]. This article is set against the backdrop of the COVID-19 pandemic reaching its peak in Hubei Province, China. Through the survey, the researchers found that during two-weeks in February 2020, health workers and business people became increasingly anxious, but anxiety among other professionals decreased. Most people in Hubei province were more positive about their risk of infection and their chances of survival. This article also reflects to some extent that the quarantine policy in the early stage of the COVID-19 outbreak did not have a great psychological impact on the Chinese people, but those medical staff who had direct contact with patients and businessmen who were affected by the quarantine were more anxious. We can learn about organizations with a high tendency to cooperate in the knowledge graph. In other words, these institutions have stronger motivations to pay attention to psychological and behavioral themes under the COVID-19 pandemic. Organizations that have published a lot of articles are mainly those with a high global reputation and have greater influence. USA, China, Italy, Australia, Spain, and Canada have performed well in national cooperation, and these countries have made leading contributions to psychological and behavioral research under the COVID-19 pandemic. We have also noticed that the countries involved in the study of highly cited papers are mainly high-income countries, especially China [20,49], the United Kingdom [50,51], Australia [52,53], and the United States [54]. Although this may be because many countries around the world regard these countries as the key forces in solving the pandemic, it also reflects a certain extent that the psychological and behavioral problems in the pandemic in low- and middle-income countries have not received enough attention [35,55]. Until the impact of the pandemic on us is reduced to a regular level, various indicators (such as the frequency of citations, the number of publications, etc.) will continue to grow, and researchers all over the world are contributing their efforts. Extensive international cooperation is necessary. Even if the pandemic is completely over, the psychological and behavioral impacts caused by this protracted battle will not end immediately, and it may even affect the next generation of people who will not experience this disaster in some ways. Children born during the pandemic have changed the way they interact with the world. Does this affect their growth? And if it does, how? Similar problems require long-term and large-scale tracking by researchers.

There are some limitations to this study. First, the term “pandemic” was not included in the search strategy. This is because there have been other pandemics in history. We know that COVID-19 broke out at the end of 2019, and the relevant publications will only have related content in 2020. But we still admit that a few publications may be missed. Secondly, our data involves research articles and reviews but does not include books and other types of publications. Many scholars’ direct response to COVID-19 is to publish articles in newspapers and magazines, or accept media interviews and express opinions on blogs. These data are not included in the presenting research. In addition, although these numbers of articles in the WoS core collection became significant in number in approximately two years, some literature not indexed by WoS were excluded from this study. Finally, the depth of the research needs to be explored further. We cannot analyze the citations yet. In the future, through citation analysis, we will be able to dig out more valuable information and provide relevant research foundations for future researchers.

## 6. Conclusions

This article used some quantitative data to strengthen and call on more researchers to pay attention to the psychological and behavioral aspects of COVID-19, because this is an area related to the well-being of the general public, and a lot of knowledge may be in the process of being formed. Through bibliometric and visualization methods, we come to the following conclusions: (1) The popularity and importance of psychological and behavioral research under COVID-19 has increased significantly, but the academic community needs to continue to pay attention to this field; (2) Related research focuses on eight hotspots, with health care workers, the elderly, students, pregnant women, family, social media, and emergency preparedness knowledge being the focus of the research object; and (3) Collaboration is relatively high at the author, organizational and national levels. However, low-income countries need to get more attention. If researchers are more concerned about how people will live after the COVID-19 pandemic and how human society will function, we believe that people who pay attention to psychology and behavior need to invest more time to consider, especially long-term follow-up research.

## Figures and Tables

**Table 1:** Database.

Indicators	Description
Database source	WoS core collection
Data indices	SCI-E, SSCI, AHCI, CPCI-S, CPCI-SSH, ESCI, CCRE, IC
Publication type	articles, review articles
Topic selection strategy	TS = (“COVID-19” OR “coronavirus disease 2019” OR “SARS-CoV-2” OR “2019-nCoV”) AND TS = (“behavio *”) AND TS = (“psycholog *”)
Record volume	2096
Record content	publication type, title, authorship, abstract, keywords, institution, country/region, journal, and citation frequency
Retrieval time	16 November 2021

Note: The data retrieved from WoS accessed from the Wuhan University library.

**Table 2:** Selected top 10 total link strength keywords.

Number	Keyword	Total Link Strength
1	mental health	931
2	anxiety	857
3	depression	673
4	stress	494
5	social distancing	308
6	lockdown	289
7	quarantine	255
8	resilience	218
9	coping	213
10	public health	203

**Table 3:** Authors of publishing at least 5 articles.

Author	*N*	Author	*N*
Griffiths, Mark D.	12	Levita, Liat	5
Mamun, Mohammed A.	10	Martinez, Anton P.	5
Lin, Chung-ying	9	Mason, Liam	5
Baeuerle, Alexander	7	Mcbride, Orla	5
Musche, Venja	7	Mckay, Ryan	5
Schweda, Adam	7	Murphy, Jamie	5
Skoda, Eva-maria	7	Shevlin, Mark	5
Teufel, Martin	7	Stocks, Thomas V. A.	5
Karekla, Maria	7	Fink, Madeleine	5
Doerrie, Nora	6	Al Mamun, Firoj	5
Kohler, Hannah	6	Hosen, Ismail	5
Weismueller, Benjamin	6	Marchetti, Daniela	5
Pakpour, Amir H.	6	Verrocchio, Maria Cristina	5
Mazza, Cristina	6	Chen, I-hua	5
Roma, Paolo	6	Ben-ezra, Menachem	5
Wang, Xueqin	6	Betsch, Cornelia	5
Yuen, Kum Fai	6	Morales, Alexandra	5
Goodwin, Robin	6	Ranjan, Piyush	5
Graffigna, Guendalina	6	Salameh, Pascale	5
Bentall, Richard P.	5	Van Der Linden, Sander	5
Hartman, Todd K.	5	Yen, Cheng-fang	5

**Table 4:** Organizations publishing at least 10 articles.

Organization Abbreviation	*N*	Organization Abbreviation	*N*
sapienza univ rome	33	univ hong kong	17
ucl	31	univ queensland	16
kings coll london	23	univ oxford	16
univ toronto	22	univ porto	16
chinese univ hong kong	22	harvard med sch	16
univ padua	22	univ new south wales	16
sun yat sen univ	20	chinese acad sci	16
univ cambrigde	19	univ michigan	15
huazhong univ sci & technol	19	harvard univ	15
nottingham trent univ	18	nyu	15
columbia univ	18	univ penn	15
natl univ singapore	18	wuhan univ	15
univ cattolica sacro cuore	18	jahangirnagar univ	15
univ milan	17		

**Table 5:** Countries and regions publishing at least 20 articles.

Country	*N*	Country	*N*
USA	496	South Korea	41
PRC	304	Saudi Arabia	39
England	245	Japan	38
Italy	228	Sweden	36
Australia	136	Russia	36
Spain	121	China Taiwan	35
Canada	112	Bangladesh	34
Germany	107	Singapore	34
India	94	Israel	32
Turkey	77	Malaysia	30
Brazil	66	Scotland	28
France	56	New Zealand	25
Netherlands	54	Austria	24
Pakistan	49	Denmark	23
Switzerland	47	Romania	23
Iran	47	Belgium	22
Poland	46	Ireland	21
Portugal	43	Norway	21

## Data Availability

According to the data access policies, the data used to support the findings of this study are available from Web of Science official website, upon a reasonable request made by email: xl.dong@hdu.edu.cn.
